# Supplementary material for: Truncated Milk Fat Globule-EGF-like Factor 8 Ameliorates Liver Fibrosis via Inhibition of Integrin-TGFβ Receptor Interaction
Source: Biomedicines. 2021 Oct 24;9(11):1529. doi: 10.3390/biomedicines9111529 (PMC8615163; doi:10.3390/biomedicines9111529)
Supplement: Supplementary file 1 [file biomedicines-09-01529-s001.zip › biomedicines-1372624-supplementary-revised.pdf]

Truncated milk fat globule-EGF-like factor 8 ameliorates liver fibrosis via inhibition of integrin-TGFβ receptor interaction.

Geun Ho An[1,2], Jaehun Lee[1], Xiong Jin[3], Jinwoo Chung[1], Joon-Chul Kim[1], Jung-Hyuck Park[1], Minkyung Kim[1], Choongseong Han[1], Jong-Hoon Kim[2,*], and Dong-Hun Woo[1,*]

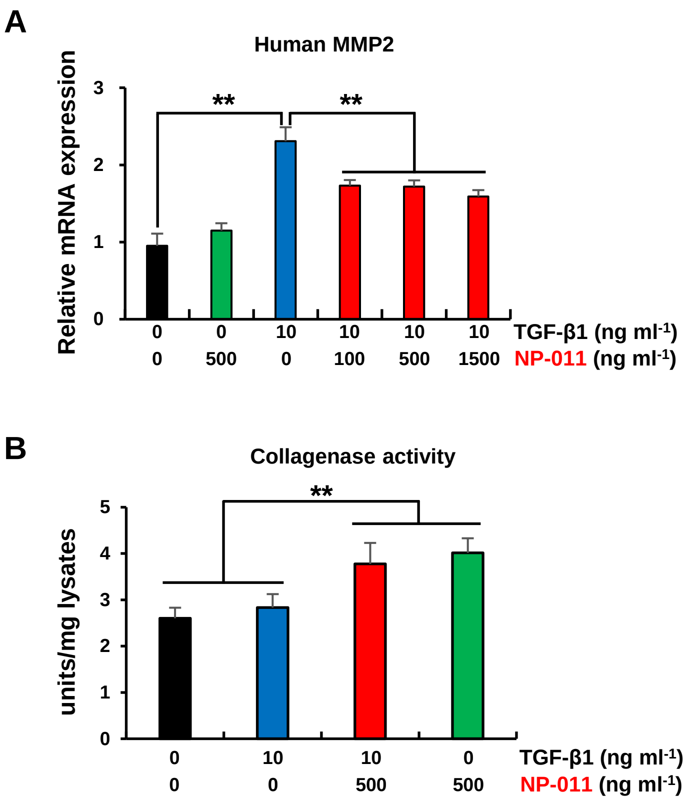

Figure S1. Regulating pro-fibrotic MMP2 and collagenase activity in HSCs. (**A**) Comparison of *MMP2* mRNA expression in human HSCs in the presence of TGF-β1, NP-011. Bars represent the means ± SD from three replicates in each group. $**P < 0.01$. (**B**) Collagenase activity assay of cell lysates from the culture of control HSCs, TGF-β1-treated HSCs with/without NP-011 treatment, and NP-011-treated HSCs. Bars represent the means ± SD from three replicates in each group. $**P < 0.01$.

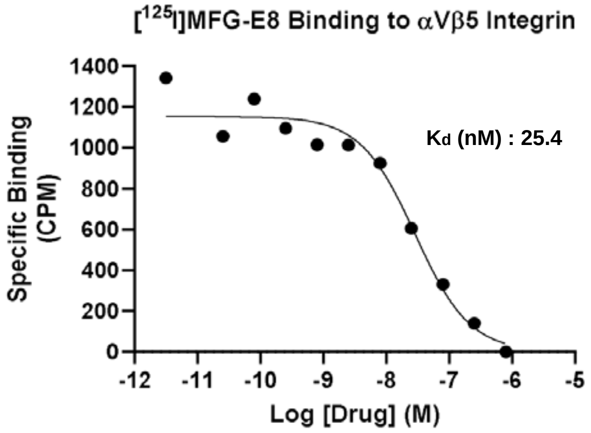

Figure S2. Radioligand binding assay to determine binding affinity of MFG-E8 to immobilized integrin αVβ5. Data was fitted using the non-linear curve fitting routines in Prism® (Graphpad Software Inc) to obtain Kd values.

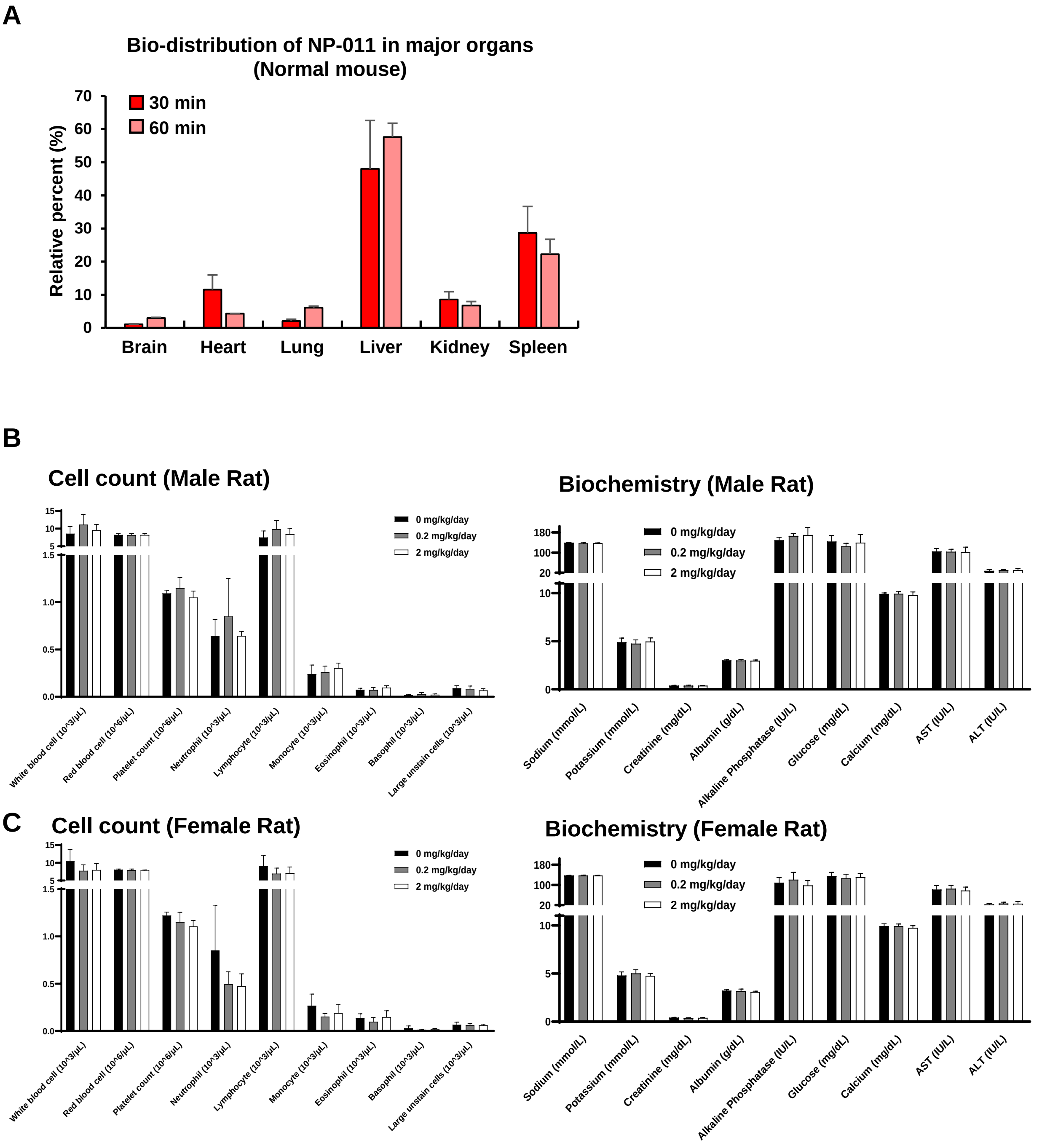

Figure S3. Biodistribution and safety profile of NP-011. (**A**) The organ distribution in healthy male mice at 30 minutes and 60 minutes after intravenously injection of NP-011 (160 μg/kg). (**B,C**) Quantitative analysis for the number of inflammatory relevant cells and blood biochemistry in male rat (**B**) and female rat (**C**) compared between normal and two NP-011 administration group.

Table S1. Primer list used in this study

Species	Primer	Sequences (5' -> 3')
Mouse	GAPDH	F: GTT GTC TCC TGC GAC TTC A
		R: GGT GGT CCA GGG TTT CTT A
Mouse	Col1a1	F: CAA TGC AAT GAA GAA CTG GAC TGT
		R: TCC TAC ATC TTC TGA GTT TGG TGA
Mouse	Col1a2	F: GCA GGG TTC CAA CGA TGT TG
		R: GCA GCC ATC GAC TAG GAC AGA
Mouse	Acta2 (α-SMA)	F: CTG ACA GAG GCA CCA CTG AA
		R: CAT CTC CAG AGT CCA GCA CA
Human	GAPDH	F: GCT CTC TGC TCC TCC TGT TC
		R: CCA TGG TGT CTG AGC GAT GT
Human	MMP2	F: AGC TCC CGG AAA AGA TTG ATG
		R: CAG GGT GCT GGC TGA GTA GAT

Table S2. Antibody list used in this study

Antibody	Vendor	Application	Designation
β-actin	Abcam	WB	ab25894
p-Smad2	Abcam	WB	ab188334
Smad2	Abcam	WB	ab33875
TGFBRI	Abcam	IP,PLA	ab31013
Integrin B3	Abcam	IP,PLA	ab25894
Integrin B5	Cell Signaling	IP,PLA	#3629
α-SMA	Santacruz	IF	sc-53142
Donkey anti-Goat IgG (H+L) Highly Cross-Adsorbed Seco ndary antibody, Alexa Fluor 594	Invitrogen	IF	A11058
Donkey anti-Rabbit IgG (H+L) Highly Cross-Adsorbed Secondary antibody, Alexa Fluor 594	Invitrogen	IF , PLA	A21207
Donkey anti-Mouse IgG (H+L) Highly Cross-Adsorbed Secondary antibody, Alexa Fluor 488	Invitrogen	IF	A21202
Goat anti-Rabbit IgG (H+L) Secondary Antibody, HRP	Invitrogen	WB	31460
Donkey anti-Sheep IgG (H+L) Secondary Antibody, HRP	Invitrogen	WB	A16041
Human MFG-E8	R&D	WB	RD27890
